# GFI1-Mediated Upregulation of LINC00675 as a ceRNA Restrains Hepatocellular Carcinoma Metastasis by Sponging miR-942-5p

**DOI:** 10.3389/fonc.2020.607593

**Published:** 2021-01-08

**Authors:** Libai Lu, Shubo Li, Ying Zhang, Zongjiang Luo, Yichen Chen, Jiasheng Ma, Pengyu Chen, Wei Wang, Jian Pu, Jianchu Wang

**Affiliations:** ^1^ Department of Hepatobiliary Surgery, Affiliated Hospital of Youjiang Medical University for Nationalities, Baise, China; ^2^ Department of Biochemistry and Molecular Biology, Youjiang Medical University for Nationalities, Baise, China; ^3^ Library of Youjiang Medical University for Nationalities, Baise, China

**Keywords:** growth factor independent 1 transcriptional repressor, lncRNA, HCC, hepatocellular carcinoma, LINC00675

## Abstract

Hepatocellular carcinoma (HCC) is a common malignant liver tumor worldwide. Tumor recurrence and metastasis contribute to the bad clinical outcome of HCC patients. Substantial studies have displayed lncRNAs modulate various tumorigenic processes of many cancers. Our current work was aimed to investigate the function of LINC00675 in HCC and to recognize the potential interactions between lncRNAs and microRNAs. GFI1 can exhibit a significant role in the progression of human malignant tumors. Firstly, GFI1 was identified using real-time PCR in HCC tissues and cells. In this work, we indicated GFI1 was remarkably reduced in HCC tissues and cells. Meanwhile, GFI1 specifically interacted with the promoter of LINC00675. Up-regulation of LINC00675 obviously repressed the migration and invasion capacity of SMCC-7721 and QGY-7703 cells *in vitro*. Moreover, decrease of LINC00675 competitively bound to miR-942-5p that contributed to the miRNA-mediated degradation of GFI1, thus facilitated HCC metastasis. The ceRNA function of LINC00675 in HCC cells was assessed and confirmed using RNA immunoprecipitation assay and RNA pull-down assays in our work. Additionally, we proved overexpression of miR-942-5p promoted HCC progression, which was reversed by the up-regulation of GFI1. In summary, LINC00675 might act as a prognostic marker for HCC, which can inhibit HCC development *via* regulating miR-942-5p and GFI1.

## Introduction

Hepatocellular carcinoma (HCC) is a frequent malignant tumor and it is the third common cause of cancer-related death across the world (1, 2). In spite of the therapeutic treatment for HCC, its survival rate is still poor because of the recurrence after surgery (3, 4). Hence, novel insights into the mechanism of HCC are in need to recognize the prognostic molecular markers to improve HCC patient survival (5).

Growth factor independent 1 transcriptional repressor (GFI1) is located in chromosome 1p22 in the human genome (6). GFI1 can act as a transcriptional repressor by interacting with other cofactors (7). It has been reported that GFI1 exhibits an important role in hematopoietic stem cells. GFI1 inhibits proliferation and preserves functional integrity in regulating self-renewal of hematopoietic stem cells (8). However, the functional role of GFI1 in HCC carcinogenesis has not been fully investigated.

LncRNAs are non-coding RNAs are longer than 200 nts, and they play few or no capacity of protein-coding (9–11). In addition, lncRNAs are dys-regulated in specific tumor types. Studies have indicated that lncRNAs can promote cancer phenotypes through interacting with DNA, RNA, and protein (12–14). Besides, it has been reported that lncRNA exhibits significant roles in HCC progression (15, 16). For example, MCM3AP-AS1 can induce the growth of HCC through regulating miR-194-5p and FOXA1 (17). LncRNA MALAT1 can contribute to HCC development through up-regulating SRSF1 and the activation of mTOR (18).

LINC00675 is also known as TMEM238L, and it has been reported to be dys-regulated in many cancers. For instance, in gastric cancer, LINC00675 is able to enhance phosphorylation of vimentin on Ser83 (19). LINC00675 represses colorectal cancer progression through sponging miR-942 and regulating Wnt/*β*-catenin signaling (20). Additionally, LINC00675 indicates short survival in patients of pancreatic ductal adenocarcinoma (21). The detailed value of LINC00675 in HCC remains unknown.

In our current study, we reported that LINC00675 repressed HCC metastasis *via* functioning as a ceRNA to reduce miR-942-5p expression level and activated the expression of GFI1. In addition, GFI1 can interact with the promoter of LINC00675 in HCC.

## Materials and Methods

### Clinical Tissues

Fifty pairs of primary human HCC cancerous tissues and corresponding adjacent liver tissues were acquired at Affiliated Hospital of Youjiang Medical University for Nationalities from 2012 to 2016. The patients given chemotherapy or radiotherapy were excluded in our work. Study approaches were approved by the Ethics Committee of Affiliated Hospital of Youjiang Medical University for Nationalities, and the informed consents were provided according to the committee regulations. Tissues were kept in liquid nitrogen upon hepatectomy immediately for future study.

### Cell Culture

Human HCC cell lines (Hep-3B, QGY-7703, SMMCC-7721, and MHCC-97L) and hepatocyte QSG-7701 cells were obtained from the Type Culture Collection of the Chinese Academy of Sciences. DMEM medium was added with 10% FBS, 100 U/ml penicillin, and 100 μg/ml streptomycin. A humidified chamber with 5% CO_2_ at 37°C was used to maintain the cells.

### Cell Transfection

The GFI siRNA and miR-942-5p mimics and inhibitors were obtained from GenePharma (Shanghai, China). Overexpression of GFI1 and LINC00675 was performed by transfection with the recombinant GFI1 and LINC00675 pcDNA3.1(+) plasmid. Lentiviral vectors for LINC00675 shRNA were constructed by Bio-Link Gene (Shanghai, China). HCC cells were transfected using lipofectamine 3000 under the official instructions.

### CCK-8 Assay

Cell viability was tested by CCK-8 kit (Dojindo, Shanghai, China). After transfection, cells were grown into 96-well plates. Then, 10 µl CCK-8 was added at various days. After 2 h, the absorbance was determined at 450 nm on a microplate reader.

### EdU Staining Assay

EdU assay was carried out using Click iT™ EdU cell proliferation assay kit (Invitrogen, Carlsbad, CA, USA). Cells were stained using 50 μM EdU for 2 h. Then, the cells were washed using PBS and fixed. Cell nuclei were stained by DAPI for 10 min. A florescence microscope was used to examine the results of cell staining.

### Flow Cytometry

Apoptosis of HCC cells was analyzed by PI and FITC Annexin V Apoptosis Detection Kit I (BD Biosciences, San Jose, CA, USA). After transfection, HCC cells were re-suspended in 1× binding buffer. Afterwards, cells were stained using 5 µl Annexin V-FITC for 15 min and 5 µl PI for 10 min. Subsequently, the apoptosis was assessed using a FACSCanto II flow cytometer.

### Cell Cycle Determination

Cells were prepared for cell suspension and washed using pre-cooled PBS. Then, cells were suspended in the mixture with 0.1 ml of pre-cooled PBS and 1 ml of pre-cooled 75% ethanol. Subsequently, cells were incubated with PI and RNase A at a final dose of 50 ng/ml. Cell cycle was determined by flow cytometry analysis.

### Cell Migration and Invasion Assay

To perform transwell migration assay, cells were seeded in the top chamber of each insert with a non-coated membrane. Then, to perform invasion assay, cells were placed in the upper chamber of each Matrigel-coated insert. Cells that migrated or invaded were fixed and stained using dye solution containing 0.1% crystal violet and 20% methanol. Afterwards, an IX71 inverted microscope (Olympus Corp, Tokyo, Japan) was used to count the cells.

### Western Blotting Analysis

HCC cells were lysed using RIPA buffer. Protein concentration was assessed using BCA protein assay kit. 30 μg protein was separated by 10% SDS-PAGE gel electrophoresis and then transferred onto a nitrocellulose membrane. Afterwards, the membranes were incubated with primary antibodies against GFI1 and GAPDH (1:1,000; Abcam, Cambridge, MA, USA). The bands were blocked with goat anti-rabbit IgG-HRP secondary antibody (1:5,000; Abcam, Cambridge, MA, USA) and were exposed by chemiluminescence substance (Pierce Biotechnology Inc., Thermo Fisher Scientific, Rockford, IL, USA).

### RT-PCR

RNA was extracted using TRIzol reagent and RNeasy Plus Micro Kit (QIAGEN, Germantown, MD, USA). Reverse transcription was carried out to synthesize the Bestar qPCR RT Kit (DBI Bioscience, Shanghai, China). Real time-PCR was carried out in Applied Biosystems 7500 Real Time PCR System (Applied Biosystems, Foster City, CA, USA) using SYBR® Green PCR Master Mix (Invitrogen, Thermo Fisher Scientific). Gene expression level was normalized to U6 RNA and GAPDH expression. Relative gene expression was evaluated using 2^−ΔΔct^. Primers were exhibited in **Table 1**.

**Table 1:** Primers for real-time PCR.

Genes	Forward (5′–3′)	Reverse (5′–3′)
GAPDH	GGAGATTGTTGCCATCAACG	TTGGTGGTGCAGGATGCATT
LINC00675	GCCTACTGCTCTGGATCATCTGGTA	ACCTGCGTCTCTTCTCCTCTTCC
GFI1 miR-942-5p U6	CCGACTCTCAGCTTACCGAG CUUCUCUGUUUUGGCCAUGUG GCTTCGGCAGCACATATACT	CTGTGTGGATGAAGGTGTGTTT CTCTACAGCTATATTGCCAGCCAC AACGCTTCACGAATTTGCGT

### *In Vivo* Assay for Metastasis

A total of 20 female BALB/c nude mice (5–6 wk old) were obtained from Beijing Wei-tong Li-hua Laboratory Animals and Technology (Beijing, China). To carry out *in vivo* metastasis assays, 3 × 10^6^ QGY-7703 cells overexpressed LINC00675 or empty vector were suspended in 300 μl serum-free DMEM per female BALB/c mice and injected through the tail vein (10 mice per group). After six weeks, mice were sacrificed and the lungs were dissected. The tissues were fixed using phosphate-buffered neutral formalin. The wax containing xenograft tissues was sliced and the xylene was utilized to do dewaxing and hydrating. The slices were stained using with H&E. Then, the slices were observed using an ECLIPSE Ti2 microscope (Ti2-U, Nikon, Tokyo, Japan). Mice were housed based on the protocols approved by the Medical Experimental Animal Care Commission of Affiliated Hospital of Youjiang Medical University for Nationalities.

### Luciferase Reporter Assay

To assess luciferase activity, Dual-Glo Luciferase Assay System (Promega, Madison, WI, USA) was performed. Lipofectamine^®^ 3000 was used to transfect cloned LINC00675/GFI1 wild-type 3′UTR or mutant 3′UTR purchased from Shanghai GeneChem (Shanghai, China) with miR-942-5p mimics, inhibitors or negative controls. 48 h later, luciferase activity was tested using the dual-luciferase reporter assay system.

### Pull Down of Biotin-Coupled miRNA

Biotin was attached to the 3′-end of miR-942-5p. Cells were transfected with miR-942-5p mimics or inhibitors using Lipofectamine 3000. Cell pellets were re-suspended in 0.7 ml lysis buffer, 0.3% NP-40, 50 U of RNase OUT, complete protease inhibitor cocktail. Then, cell lysate was isolated by centrifugation at 10,000 g. Finally, the level of LINC00675 or in the pull down of biotin-miR-942-5p was quantified using real-time PCR.

### Statistical Analysis

The data was analyzed using GraphPad Prism 6.0 and SPSS 22.0. Student’s t-test was employed to compare two groups and differences among more than two groups were compared by one-way ANOVA. A value of p <0.05 was considered to be statistically significantly.

## Results

### GFI1 Is Down-Regulated in HCC Patients and Tissues

**Figure 1:**
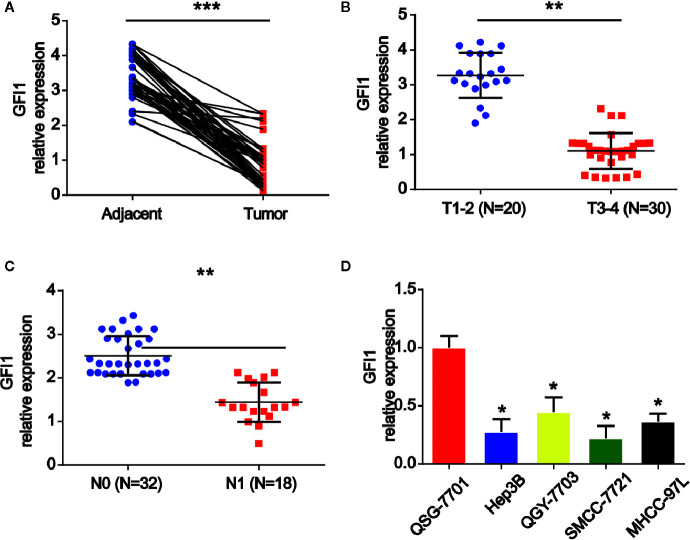
GFI1 was increased in HCC tissues and HCC cells. **(A)** The expression of GFI1 was detected by real-time PCR in in 50 pairs of HCC carcinoma and normal adjacent tissues. **(B)** Expression of GFI1 in HCC tissues at different stages. **(C)** Expression of GFI1 in HCC tissues with metastasis and without metastasis. **(D)** Expression of GFI1 in HCC cells (Hep-3B, QGY-7703, SMCC-7721 and MHCC-97L) and QSG-7701 cells. *P < 0.05; **P < 0.01; ***P < 0.001.

Firstly, we investigated the expression of GFI1 in HCC tissue samples and adjacent tissues. It was shown that GFI1 was obviously reduced in HCC tissues as displayed in **Figure 1A**. In **Figure 1B**, it was indicated that GFI1 was decreased in advanced HCC tissues (stages T3–T4) compared to T1–T2 stages. In addition, in **Figure 1C**, we found that GFI1 LINC00675 was greatly decreased in HCC tissues with lymphatic metastasis compared. Next, we confirmed that GFI1 was also decreased in HCC cells (Hep3B, QGY-7703, SMCC-7721 and MHCC-97L) compared with the QSG-7701 cells in **Figure 1D**.

### LINC00675 Is Modulated by the Transcription Factor GFI1

**Figure 2:**
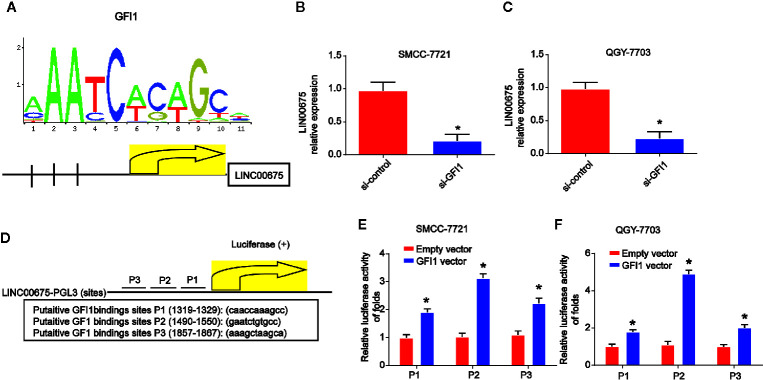
GFI1 acted as a transcription inducer of LINC00675. **(A)** DNA motif of GFI1 in the promoter of LINC00675. **(B, C)** Expression of GFI1 in QGY-7703 and SMCC-7721 cells transfected with siRNA of GFI1 for 48 h. **(D)** The predicted three binding sites of GFI1 in LINC00675 promoter. **(E, F)** Luciferase activity analysis of the binding sites in HCC cells transfected with GFI1 oligonucleotides. *P < 0.05.

In order to study the mechanism of LINC00675 down-regulation in HCC, bioinformatical software program JASPAR (http://jaspar.genereg.net/cgi-bin/jaspar_db.pl) was carried out to analyze the promoter regions of LINC00675, and three potential sites of GFI1 binding were predicted. In **Figure 2A**, the DNA motif of GFI1 in UBE4B promoter was demonstrated. QGY-7703 and SMCC-7721 cells were transfected with GFI1 siRNA. LINC00675 expression was significantly decreased after GFI1 was reduced in HCC cells (**Figures 2B, C**). The LINC00675 promoter region including three binding sites of GFI1 was inserted into a PGL3 vector as displayed in **Figure 2D**. As shown in **Figures 2E, F**, GFI1 significantly enhanced the luciferase activity in HCC cells.

### Up-Regulation of LINC00675 Represses HCC Cell Proliferation, Migration, and Invasion

**Figure 3:**
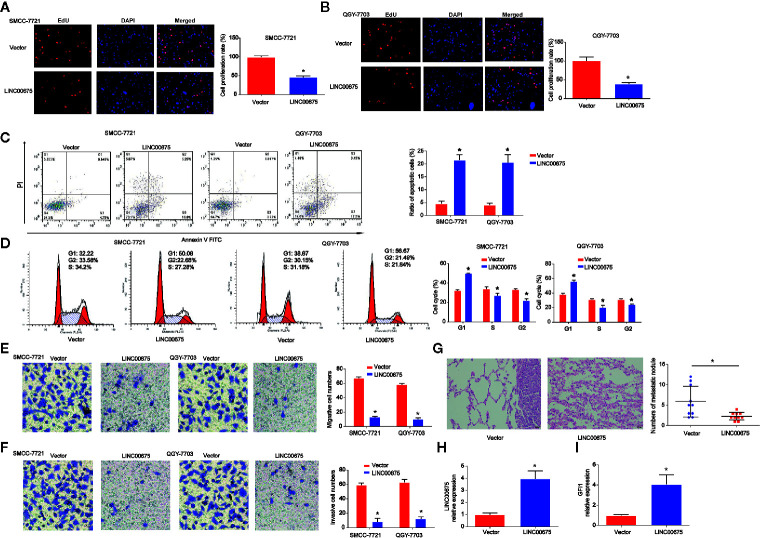
Effects of LINC00675 on HCC cell proliferation, migration, and invasion. **(A, B)** EdU assay was carried to test cell viability. QGY-7703 and SMMC-7721 cells transfected with LINC00675 overexpression vector. **(C, D)** Effects of LINC00675 on HCC cell apoptosis and cell cycle. **(E, F)** Effects of LINC00675 on HCC cell migration and invasion. Transwell migration and invasion assay was carried out to evaluate cell migration and invasion capacity. **(G)** Gross morphology of representative lungs and characteristic H&E staining of metastatic nodules in the lung of nude mice. **(H, I)** LINC00675 and GFI1 expression in lung tissues. n = 10 mice per group. *P < 0.05.

Moreover, to study the effect of LINC00675 on HCC cell proliferation, QGY-7703 and SMCC-7721 cells were transfected with LINC00675 overexpression plasmid. EdU assay indicated that overexpression of LINC00675 significantly inhibited QGY-7703, SMCC-7721 cell proliferation compared with the control groups in **Figures 3A, B**. As shown in **Figure 3C**, QGY-7703 and SMCC-7721 cell apoptosis was obviously triggered by the overexpressed LINC00675. In addition, in **Figure 3D**, HCC cell cycle distribution was blocked in G1 phase significantly by LINC00675. Transwell migration and invasion assay implied that the migrated and invaded cells in LINC00675 overexpression groups were notably less than the cells in the LV-NC group in **Figures 3E, F**. Then, we implanted either control or QGY-7703 cells with stable overexpressed LINC00675. In **Figure 3G**, it was shown that LINC00675 overexpression significantly depressed the number of metastatic lung nodules. Subsequently, we confirmed that LINC00675 and GFI1 expression was greatly increased in mice lung tissues (**Figures 3H, I**).

### LINC00675 Abundantly Sponges miR-942-5p

**Figure 4:**
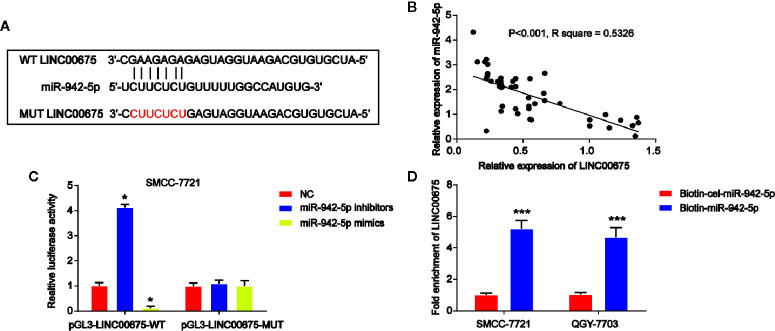
LINC00675 sponged miR-942-5p in HCC cells. **(A)** The putative binding sites between miR-942-5p and LINC00675 and the mutant sites in LINC00675-MUT reporter were displayed. **(B)** Correlation between miR-942-5p and LINC00675 in 50 pairs of HCC tissues. **(C)** Luciferase activity was evaluated in SMCC-7721 cells co-transfected with WT-LINC00675 or MUT-LINC00675 reporter and miR-942-5p inhibitors or mimics. **(D)** LINC00675 was pulled down by biotinylated wild-type miR-942-5p. *P < 0.05, ***P < 0.001.

Then, by using bioinformatics analysis, the binding sites between LINC00675 and miR-942-5p was exhibited in **Figure 4A**. Luciferase reporter plasmids of WT-LINC00675 and MUT-LINC00675 binding sites were displayed in **Figure 4A**. In addition, a negative correlation between LINC00675 and miR-942-5p was shown in HCC tissues (**Figure 4B**). Co-transfection of the WT-LINC00675 with miR-924-5p inhibitors induced the reporter activity while the mimics reduced the reporter activity (**Figure 4C**). In **Figure 4D**, LINC00675 was most abundantly pulled down by miR-942-5p in QGY-7703 and SMCC-7721 cells.

### Loss of miR-942-5p Restrains HCC Cell Growth, Migration, and Invasion Induced by Loss of LINC00675

**Figure 5:**
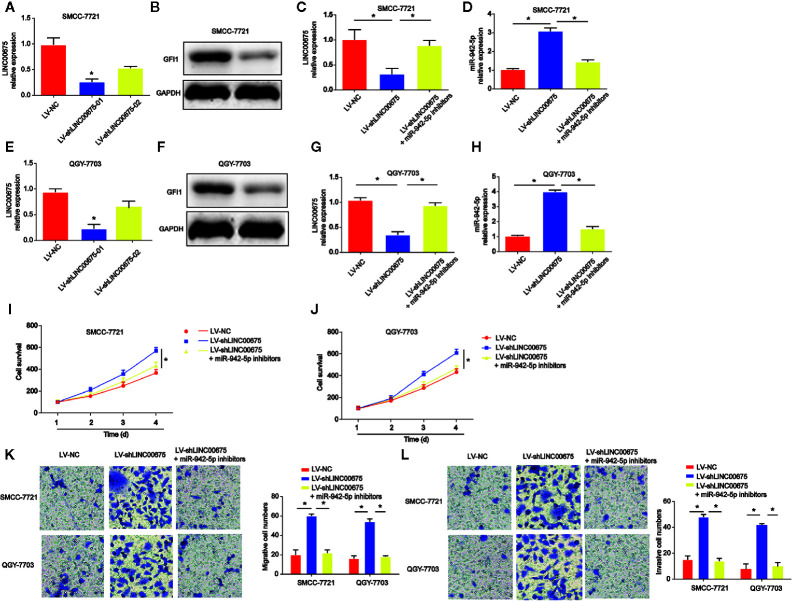
Effects of miR-942-5p on HCC cell proliferation, migration, and invasion. **(A)** Expression of LINC00675 in SMMC-7721 cells transfected with LINC00675 shRNA. **(B)** Expression of GFI1 protein in SMMC-7721 cells transfected with LINC00675 shRNA. **(C)** Expression of LINC00675 in SMMC-7721 cells transfected with LV-shLINC00675 and miR-942-5p inhibitors. **(D)** Expression of miR-942-5p in SMMC-7721 cells transfected with LV-shLINC00675 and miR-942-5p inhibitors. **(E)** Expression of LINC00675 in QGY-7703 cells transfected with LINC00675 shRNA. **(F)** Expression of GFI1 protein in QGY-7703 cells transfected with LINC00675 shRNA. **(G)** Expression of LINC00675 in QGY-7703 cells transfected with LV-shLINC00675 and miR-942-5p inhibitors. **(H)** Expression of miR-942-5p in QGY-7703 cells transfected with LV-shLINC00675 and miR-942-5p inhibitors. **(I, J)** Effects of miR-942-5p on HCC cell proliferation. **(K, L)** Effects of miR-942-5p on HCC cell migration and invasion. *P < 0.05.

In **Figures 5A, E**, SMCC-7721 and QGY-7703 cells were transfected with LINC00675 shRNA. LINC00675-01 exhibited a better knockdown effect, and it was chosen for the following assays. In addition, GFI1 protein expression was significantly reduced by loss of LINC00675 shRNA in **Figures 5B, F**. Then, SMCC-7721 and QGY-7703 were transfected with miR-942-5p inhibitors after loss of LINC00675. As shown in **Figures 5C, G**, LINC00675 was greatly reduced by LINC00675 shRNA and inhibitors of miR-942-5p induced LINC00675 expression. For another, we observed that miR-942-5p was increased after LINC00675 was increased in QGY-7703 and SMCC-7721 cells, which was successfully decreased by the inhibitors (**Figures 5D, H**). Furthermore, HCC cell proliferation, migration and invasion capacity was obviously enhanced by loss of LINC 00675, which was repressed by miR-942-5p inhibitors (**Figures 5I–L**).

### GFI1 Is a Downstream Target of miR-942-5p

**Figure 6:**
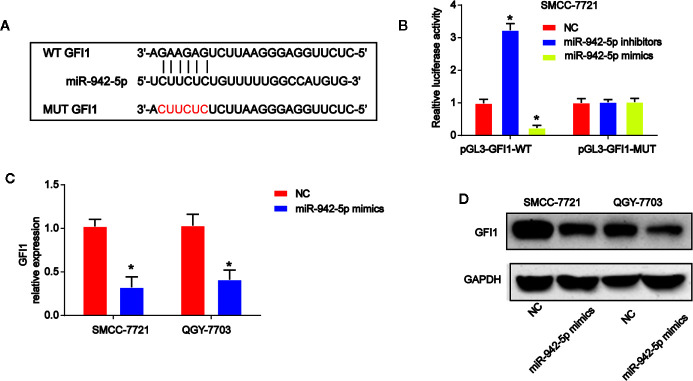
miR-942-5p targeted GFI1 in HCC cells. **(A)** The putative binding sites between miR-942-5p and GFI1 and the mutant sites in GFI1-MUT reporter were displayed. **(B)** Luciferase activity was evaluated in SMCC-7721 cells co-transfected with WT-GFI1 or MUT-GFI1 reporter and miR-942-5p inhibitors or mimics. **(C, D)** GFI1 mRNA and protein expression in SMCC-7721 cells transfected with miR-942-5p mimics. Error bars stand for the mean ± SD of at least triplicate assays. *P < 0.05.

Moreover, by using bioinformatics analysis, the binding sites between GFI1 and miR-942-5p was displayed in **Figure 6A**. Luciferase reporter plasmids of WT-GFI1 and MUT-GFI1 binding sites were demonstrated in **Figure 6A**. Co-transfection of the WT-GFI1 with miR-924-5p inhibitors enhanced the reporter activity while the mimics of miR-924-5p inhibited the reporter activity (**Figure 6B**). In addition, miR-924-5p mimics reduced GFI1 mRNA, and protein expression significantly in QGY-7703 and SMCC-7721 cells (**Figures 6C, D**).

### Overexpression of GFI1 Depresses HCC Cell Progression Triggered by miR-924-5p Mimics

**Figure 7:**
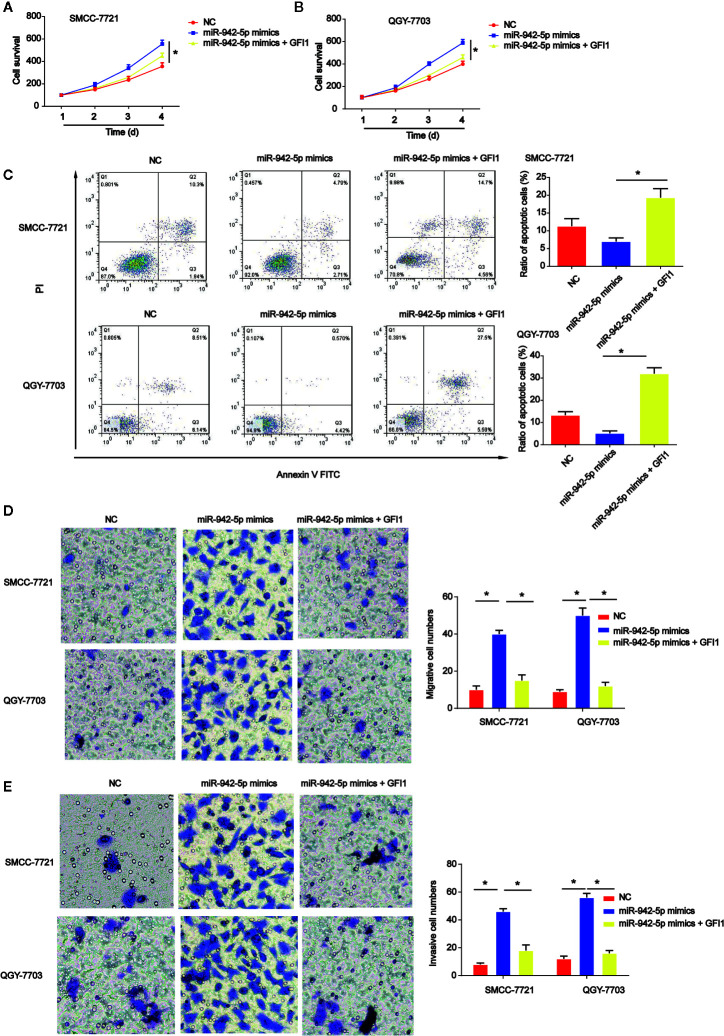
Effects of GFI1 on HCC cell progression was reversed by miR-942-5p mimics. **(A, B)** HCC cell survival was tested using EdU assay. HCC cells were transfected with miR-942-5p mimics and GFI overexpression plasmid. **(C)** Effects of GFI1 on HCC cell apoptosis. **(D, E)** Effects of GFI1 on HCC cell migration and invasion. *P < 0.05.

Subsequently, QGY-7703 and SMCC-7721 cells were transfected with GFI1 overexpression plasmid after miR-942-5p mimics were transfected into the cells. CCK-8 indicated that HCC cell survival was increased by miR-942-5p overexpression, which was reversed by GFI1 overexpression as shown in **Figures 7A, B**. In **Figure 7C**, we found that HCC cell apoptosis was increased by the up-regulation of GFI1. Additionally, QGY-7703 and SMCC-7721 cell migration and invasion were increased by the mimics of miR-942-5p, which was decreased by increased GFI1 as manifested in **Figures 7D, E**.

## Discussion

In our present work, we identified LINC00675 was aberrantly expressed in human HCC tissues and cells. As known, lncRNAs with differential expression in malignant tumors can indicate the prognosis (22–24). A growing number of biomarkers are reported in patients with HCC (25). For example, lncRNA-D16366 has been identified as a potential biomarker for HCC (26). Down-regulation of lncRNA ZNF385D-AS2 expression exhibits a crucial prognostic significance in HCC (27).

The data of our present study displayed that GFI1/LINC00675/miR-942-5p as a prognostic factor in HCC development. GFI1 was frequently reduced in HCC tissues and HCC cell lines. Then, we focused on the potential mechanism responsible for LINC00675 down-regulation in HCC cells. GFI1 could interact with the LINC00675 promoter through the three binding sites. Our current results confirmed the regions on the LINC00675 promoter. miR-942-5p was predicted as a downstream target of LINC00675. Loss of LINC00675 induced HCC cell growth, which was reversed by down-regulation of miR-942-5p. Additionally, GFI1 acted as a direct target for miR-942-5p.

LINC00675 is a kind of intergenic lncRNA, and increasing studies demonstrates LINC00675 is dys-regulated in various cancers. For instance, LINC00675 can regulate cervical cancer cell growth through affecting Wnt/*β*-catenin signaling (28). LINC00675 can repress tumorigenesis and EMT of esophageal squamous cell carcinoma *via* repressing Wnt/*β*-catenin pathway (29). In addition, LINC00675 is a significant prognostic factor of glioma, which can regulate cell proliferation, migration and invasion (30). These findings support the potential tumor inhibitory role of LINC00675 in cancers. Here, we observed that LINC00675 was decreased in HCC and up-regulation of LINC00675 significantly repressed HCC cell proliferation, migration and invasion. Then, the detailed mechanism of LINC00675 in regulating HCC progression was investigated.

Transcription factors exhibit crucial roles in determining cell fate and behavior. GFI1 is a kind of DNA binding zinc finger protein and it can mediate transcriptional repression through recruiting histone-modifying enzymes to its targets (31). Down-regulation of GFI1 can promote inflammation-linked metastasis of colorectal cancer, and it is a tumor suppressor gene (32, 33).Currently, we proved that GFI1 was down-regulated in HCC tissues and cells. Meanwhile, GFI1 interacted with the LINC00675 promoter *via* the three binding sites. The binding sites on the LINC00675 promoter contained regulatory elements for the transcription of LINC00675 were exhibited. Additionally, whether GFI1 could serve as a downstream target of LINC00675 was explored.

As well known, lncRNAs can act their roles *via* sponging microRNAs to modulate the target gene expression (34). microRNAs are small non-coding RNAs to repress gene expression through binding to the 3′-UTR of target mRNAs (35). For another, miR-942-5p was predicted as the target for LINC00675. Previously, it has been shown that lncRNA LIFR-AS1 can inhibit invasion and metastasis of lung cancer *via* regulating miR-942-5p and ZNF471 (36). Additionally, miR-942-5p is sequestered by circRNA-AKT1 to induce AKT1 and contributes to cervical cancer progression (37). In the present study, we proved miR-942-5p can interact with LINC00675, and GFI1 acted as a downstream target for miR-942-5p. Thus, LINC00675 could regulate GFI1 expression indirectly *via* sponging miR-942-5p.

In summary, we reported GFI1 was decreased in HCC and it can modulate LINC00675 expression positively. Loss of LINC00675 might serve as a tumor inhibitor in HCC cell growth and progression *via* sponging miR-942-5p.

## Data Availability Statement

The original contributions presented in the study are included in the article/supplementary material. Further inquiries can be directed to the corresponding author.

## Ethics Statement

The studies involving human participants were reviewed and approved by Affiliated Hospital of Youjiang Medical University for Nationalities. The patients/participants provided their written informed consent to participate in this study. The animal study was reviewed and approved by Affiliated Hospital of Youjiang Medical University for Nationalities.

## Author Contributions

JW designed the research and revised the manuscript. LL, SL and YZ performed the experiments. ZL and YC collected the data. JM and PC did the analysis. WW and JP supported the study. LL drafted the manuscript. All authors contributed to the article and approved the submitted version.

## Funding

This work was supported by the Grants from Science and Technique Research Projects of Guangxi, No. 2019JJA140524.

## Conflict of Interest

The authors declare that the research was conducted in the absence of any commercial or financial relationships that could be construed as a potential conflict of interest.
